# Effects of physical exercises on semen quality and reproductive outcomes in male infertility: A protocol for systematic review and meta-analysis of randomized controlled trials

**DOI:** 10.1097/MD.0000000000017494

**Published:** 2019-10-11

**Authors:** Xuhong Yan, Liang Dong, Yinghong Liu, Fang Yang, Kun Tan, Junjun Li, Degui Chang, Xujun Yu

**Affiliations:** a Hospital of Chengdu University of Traditional Chinese Medicine; b Department of Andrology, The Reproductive & Women-Children Hospital, Chengdu University of Traditional Chinese Medicine; c School of Sports Medicine and Health Chengdu Sport institute, Chengdu Sport University, Chengdu, Sichuan, China.

**Keywords:** live birth, physical exercise, pregnancy rate, reproductive outcome, semen quality, systematic review

## Abstract

**Background:**

Infertility has troubled the world's 186 million people, and male infertility accounts for more than half. The literature of physical exercise related to semen quality has shown inconsistent results, and there is currently no systematic review to evaluate the effects of exercise on reproductive outcomes in male infertility patients. This study aims to assessing the effects of exercise interventions based on randomized controlled trials (RCTs) on semen quality and reproductive outcomes in male infertility.

**Methods:**

English and Chinese literature about physical exercise treatment for male infertility published before July 31, 2019 will be systematic searched in PubMed, Embase, Web of Science, Cochrane Library, Open Grey, Clinicaltrials.gov, Chinese Clinical Trial Registry, WANFANG, VIP Chinese Science and Technology Journal Database, CNKI, Chinese biomedical document service system (SinoMed). Only RCTs of patients with male infertility will be included. Literature screening, data extraction, and the assessment of risk of bias will be independently conducted by 2 reviewers, and the 3rd reviewer will be consulted if any different opinions existed. Live-birth rate, pregnancy rate, adverse events (including miscarriage), sperm concentration, progressive motility, sperm morphology, and sperm DNA fragmentation will be evaluated. Systematic review and meta-analysis will be produced by RevMan 5.3 and Stata 14.0. This protocol reported in accordance with the Preferred Reporting Items for Systematic Review and Meta-analysis Protocols (PRISMA-P) statement, and we will report the systematic review by following the PRISMA statement.

**Conclusion and dissemination:**

We will assess the efficacy and safety of physical exercise on semen quality and reproductive outcomes in infertile men. The findings will be published in a peer-reviewed journal to provide evidence-based medical evidence for clinical decision making and the patient's lifestyle guidance.

**Registration information:**

PROSPERO CRD42019140294

## 1 Introduction

Infertility refers to the failure to obtain a successful pregnancy after 12 months or longer of appropriate and timed unprotected intercourse,^[1]^ impacts an estimated 15% of couples globally.^[2]^ About 186 million infertile patients worldwide, and more than half of them are male infertility cases.^[3]^

There are many reasons for male infertility. Sedentary work can double the high risk of sperm DNA damage.^[4]^ Lack of exercise and sedentary can affect infertility.^[5]^ In addition, oxidative stress is a common cause of male infertility,^[6,7]^ and the rise of inflammatory cytokines in seminal plasma is closely related to sperm quality decline^[8]^ and sperm DNA damage.^[9]^

A randomized controlled trial (RCT) showed that resistance exercise^[10]^ may regulate male infertility by anti-inflammatory factors and antioxidant mechanisms. Recently, new guidelines for male oxidative stress infertility suggest that evidence-based lifestyle management against oxidative stress reasons should be provided, including exercise.^[7]^

Whether exercise improves semen quality is still a controversial point of view. An observational study showed that semen parameters were not associated with routine physical activity, and that cycling over 5 hours per week was associated with decreased sperm concentration and total sperm motility.^[11]^ RCTs have shown that physical exercise can improve semen parameters^[12,13]^ and live-birth outcomes^[13]^ in male infertility. A recent systematic review which included normal male population, observational and interventional studies^[14]^ showed that elite sports were harmful to sperm motility but recreational physical activities may improve semen quality. Interestingly, compared with infertile men, healthy people may have more normal semen parameters. Therefore, the sensitivity of semen parameters to the intervention of exercise may be inconsistent between infertile men and healthy people. Besides, RCTs and their systematic reviews provide the most reliable evidence of medical intervention,^[15]^ and the quality of evidence is higher than that of observational studies.^[16]^ Since there is currently no systematic review of exercise intervention for treatment of male infertility based on RCTs, it is necessary to conduct this systematic review.

### 1.1 Review objectives

The objective of this research is assessing the effects of exercise on infertile male population including live-birth rates, pregnancy rates and adverse events (including miscarriage), sperm DNA fragmentation, progressive motility, sperm concentration, sperm morphology. The results of the research will provide evidence for andrologists in decision-making and lifestyle guidance.

## 2 Methods

This is a systematic review and will be a meta-analysis if necessary. Since the data and results used are all from published studies, no ethics committee approval is required.

### 2.1 Protocol and registration

We registered this study on Prospero. Registration number: CRD42019140294.

This protocol refers to the statement of Preferred Reporting Items for Systematic Review and Meta-analysis Protocols (PRISMA-P).^[17,18]^ And we will report the systematic review by following the PRISMA statement.

### 2.2 Data source

#### 2.2.1 Electronic search database and approach

We will search in PubMed, Embase, Cochrane library, Web of science, China National Knowledge Infrastructure (CNKI), WANFANG DATA, Chinese biomedical document service system (SinoMed), VIP Chinese Science and Technology Journal Database (VIP). Only English and Chinese languages will be used for search. We will search from the establish date to July 31, 2019 of each platform or database. The searching work will be done in August 2019 and updated before the systematic review has completed.

Subject heading, lower words, entry terms, and free words search will be used in PubMed, Embase, and Cochrane library. Cochrane library search will be restricted by using “search word variations.” Topic search will be used in Web of Science. Free words will be searched within title, abstract, keywords in Cochrane library, Embase and within title, and abstract in PubMed. Chinese database search: CNKI will be restricted by using “topic” field; WANFANG and VIP will be limited by “title or keyword” filed; SinoMed will be searched by using subject words search plus synonym retrieval.

Search terms include: “male infertility” or “male subfertility” AND “exercise” or “sports” or “exercise movement techniques” or “exercise therapy” or “physical activity” AND “pregnancy rate” or “live birth” or “semen” or “sperm.” Chinese search will use the Chinese form of the above terms. The example of specific search for PubMed is shown in Table 1.

**Table 1:**
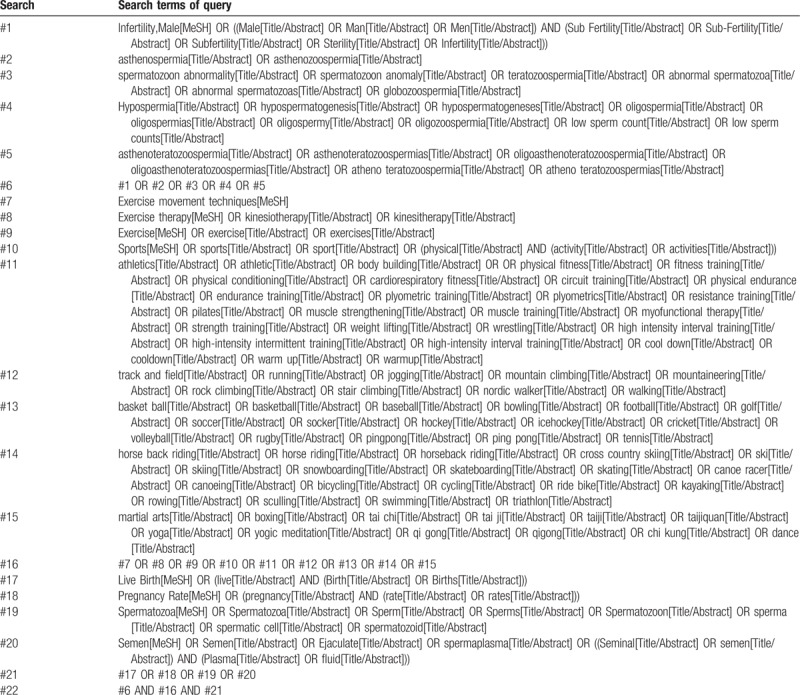
PubMed search strategies.

#### 2.2.2 Other sources of search

Clinicaltrials.gov will be searched for ongoing or unpublished clinical trials. Gray literature will be retrieved through OpenGrey. Library interlibrary loan and Baidu academic will be used to assist in the acquisition of full-text documents, and references from exist systematic reviews and meta-analyses that are detected will also be retrieved.

### 2.3 Included and excluded criteria

#### 2.3.1 Study design

This study will include only RCTs. Other studies such as observational studies, retrospective analyses, self-controlled trials, patient series, case reports, reviews, animal studies, and laboratory in vitro studies will be excluded.

#### 2.3.2 Participants

##### 2.3.2.1 Included population

Men who meet the inclusion criteria should be diagnosed as male factor infertility, aged 18 to 60 years, and their couples may or may not receive assisted reproductive technology, including intrauterine insemination (IUI), in vitro fertilization (IVF), and intracytoplasmic sperm injection (ICSI). The diagnosis of male infertility should be in accordance with the European Urological Association's 2012 edition^[19]^ or other authoritative standards.

##### 2.3.2.2 Excluded population

The exclusion criteria were healthy people; infertility with female factors; azoospermia; infertility caused by obstruction, hypothalamic-pituitary lesions, congenital abnormality, endogenous or exogenous hormone abnormalities.

#### 2.3.3 Interventions

Intervention in the intervention group must be exercise. The type of exercise is 1 physical activity or a combination of more than 1 physical activity. The intervention in the control group can be sedentary or no physical exercise intervention. Studies with drug therapy in the intervention group and control group will be excluded.

##### 2.3.3.1 Definition of exercise

In this research, exercise defined as physical activity, sport, or exercise therapy used to improve or maintain physical fitness or health, which includes the following large categories:

1. Traditional oriental fitness exercise: These activities usually require a combination of breath or mind adjustment during body exercise such as Tai Chi, Qigong, yoga, etc.
2. Competitive sports: These sports usually conduct by professional athletes for the purpose of competition such as gymnastics, track and field, ball, swimming, weightlifting, etc.
3. Other activities: All sports except the above 2 large categories such as outdoor or indoor aerobic exercise, resistance training, etc.

##### 2.3.3.2 Classification of exercise intensity

Exercise intensity is divided into 5 categories according to the Position statement on physical activity and exercise intensity terminology.^[20]^ The specific definition and classification criteria are shown in Table 2.

**Table 2:**
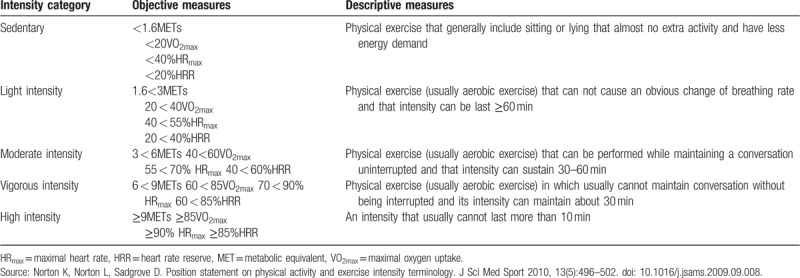
Classification of exercise intensity and objective measurements and descriptive measurements of each intensity.

#### 2.3.4 Outcomes

##### 2.3.4.1 Primary outcome indicator

Live-birth rate refers to all live birth of successful pregnant couples or all live birth reported in the study.

##### 2.3.4.2 Secondary outcome indicators

1. Pregnancy rate: defined as all pregnancy reported in the study.
2. Adverse events (including miscarriage)^[21]^: refers to miscarriage, injuries during exercise, or other adverse events reported in the study.
3. Sperm DNA fragmentation: abnormal DNA integrity reported in the study. The detection method may be sperm chromatin structure assay, terminal deoxyuridine nick end-labeling (TUNEL) assay, Comet assay, sperm chromatin dispersion assay, Acridine orange test, Aniline blue staining, Toluidine blue, and Chromomycin A3 staining.^[22]^
4. Sperm concentration: defined as the number of sperm (×10^6^/mL).^[21]^
5. Progressive motility sperm: including A+B or forward-moving sperm in the World Health Organization classification, provided as a percentage (%).
6. Sperm morphology: provided as a percentage (%).^[21]^

### 2.4 Selection of studies and data extraction

Endnote X8 software will be used to manage and screening literature. Document screening and data extraction will be independently performed by 2 professionally trained reviewers (XH Y, LD).

The software will be used for repeated literature screening at 1st, then the title and abstract will be read, and the duplicated literature will be removed.

Further screening the literature based on inclusion and exclusion criteria. In this process, the controversial literature will be screened after the full text is obtained.

**Figure 1:**
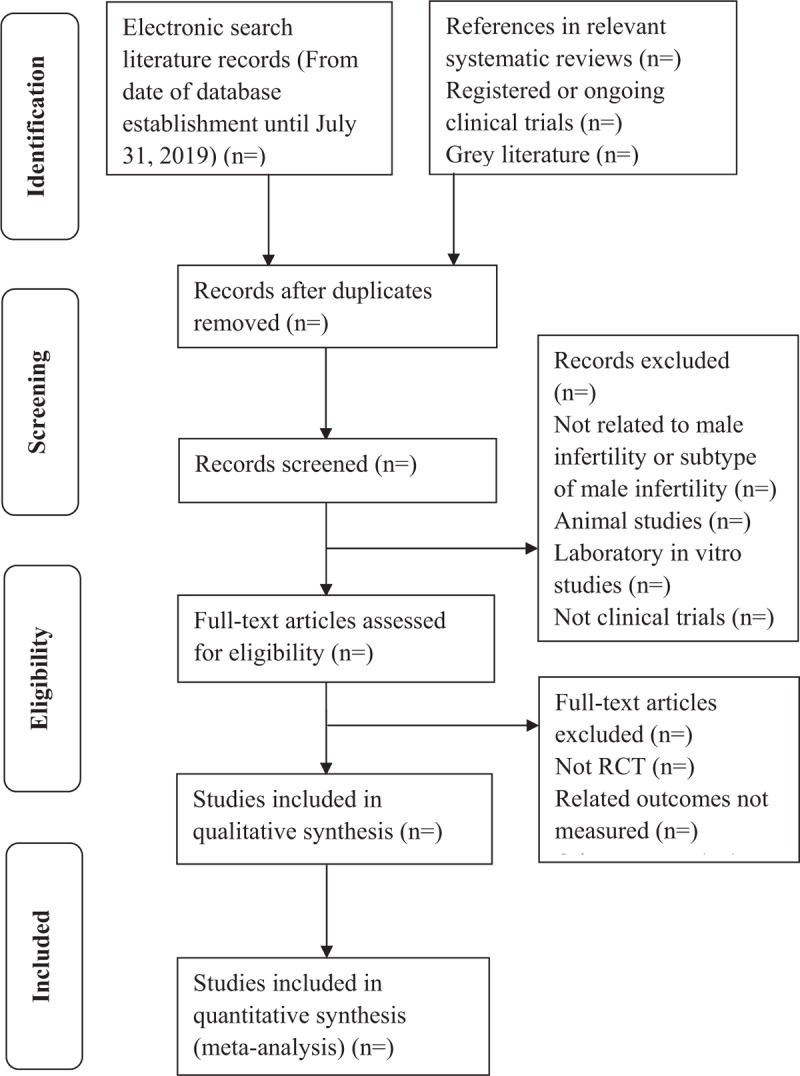
The Preferred Reporting Items for Systematic Review and Meta-analysis Protocols (PRISMA) literature screening flow chart.

Finally, the full-text reading and screening of the articles that meet the inclusion criteria will be carried out. If 2 or more articles have duplicate or phased study results, only the ones with the largest sample size and the most complete intervention and follow-up time are included. When the review group judges that there may be duplicate studies but cannot confirm them, the original research author will be contacted for judgment. The literature screening flow chart is shown in Figure 1.

Before data extraction, the whole group will discuss and produce a unified data extraction table. Two reviewers (XH Y, LD) will conduct data extraction exercises, the data of 3 to 4 articles will be extracted to detect data extraction consistency and the integrity of the table. The contents of data extraction are as follows:

1. General characteristics of studies: 1st author, year of publication, title, region and country, execution time of study, corresponding author, contact information.
2. Methodology and design of studies: study design, sample size, sample size of subspecies, randomized information, assignment hiding, blind method, diagnostic criteria, outcome indicators, safety indicators, statistical methods, supervision, method and measurement time of outcome indicators, methods, and time of follow-up.
3. Information of participants/patients: age, race, severity of disease, course of disease, baseline level, diagnosis or subtype of disease, comorbidity, and spouse's healthy condition.
4. Information of intervention and control measures: name, type, intensity, duration, combination, phase, frequency, execution time, place, and other combined treatments (including IUI, IVF, and ICSI).
5. Data of outcome indicators: results of each outcome indicator at different measurement time points, data of adverse events, and specific information.
6. Data for risk of bias: random sequence generation, allocation concealment, blinding of participants and personnel, incomplete outcome data, selective reporting, and other bias.^[15]^
7. Other information: source of funding and conflict of interest.

During the data extraction process, all disagreement will be solved after discussion with the 3rd reviewer (YL). If there are abstracts of the conference papers meeting the inclusion criteria, the review group will contact the original research author by email to obtain the full text or related results. If there is any question or confuse about the original research in data extraction process, we will contact the author of the original research by email to obtain specific answers.

### 2.5 Risk of bias assessment

Two authors (XH Y, LD) will independently assess the risk of bias based on the Cochrane Collaboration's tool for assessing risk of bias.^[15]^ The disagreement will be decided through discussing with the 3rd author (YL). Assessment items include random sequence generation, allocation concealment, blinding of participants and personnel, blinding of outcome assessment, incomplete outcome data, selective reporting, and other bias.^[15]^ Each item of bias situation includes low risk, unclear, and high risk.^[15]^ Since the studies of exercise is not convenient for blinding, the outcome indicators of this systematic evaluation are relatively objective, so we define the generation of random sequence, allocation concealment and incomplete data as key domains of risk of bias evaluation. The risk of bias assessment chart of inclusion studies will be produced by using Review Manager 5.3 software.

### 2.6 Data analysis and synthesis

Data analysis and synthesis will be performed with reference to 2 books on evidence-based medicine.^[23,24]^ When the data of outcomes cannot be extracted or significant clinical heterogeneity exist in included trials, descriptive analysis or narrative synthesis will be adopted. When the included trials are homogeneous in clinical and the data available, meta-analysis will be implemented. Heterogeneity and the size of heterogeneity will be test by Cochrane *Q* test and *I*^2^ statistic, respectively. The heterogeneity exists when *P* ≤ .10. If *I*^2^ ≤ 50%, the heterogeneity between multiple studies is acceptable. And the fixed effect model will be adopted to calculate the effect size of risk ratio (RR) by Mantel–Haenszel method and mean difference (MD) by inverse variance. If *I*^2^ > 50%, there is a high heterogeneity among multiple studies. In this case, the causes of heterogeneity such as the method and intensity of intervention, duration of treatment, severity of disease, subtype of disease, and whether the control measures are the same will be analyzed at 1st. Subgroup analysis will be used according to the specific situation afterwards. If the results of multiple similar studies are still heterogeneous after being processed by the above methods, the random effects model (DerSimonian & Laird method) will be used to calculate the effect size. The effect size of numerical variables will be expressed by MD and 95% confidence interval (CI), and the dichotomous variable effect size will be expressed by RR and 95% CI. A *P*-value ≤.05 of *Z*-test for effect size will be considered as statistically significant. Meta-analysis will be produced by Review Manager 5.3 and be presented as forest plot.

### 2.7 Subgroup analysis

Subgroup analysis will be performed according to age, ethnicity, different subtypes of male infertility disease, and duration of intervention.

### 2.8 Sensitivity analysis

Sensitivity analysis will be used to detect the stability, reliability of the results of meta-analysis and to detect the sources of heterogeneity. It will be performed by excluding the trials with high risk of bias or eliminating each study one by one. The meta-analysis will then be performed again and the results compared to the previous meta-analysis.^[23,24]^

### 2.9 Publication bias

The publication bias will be tested by the Egger test (Stata software 14.0). When it includes more than 10 trials, the funnel plot (Review Manager 5.3 software) will be used to detect publication bias.^[23,24]^

## 3 Discussion

At present, male infertility treatment methods are diverse, including antioxidants^[7,21]^ and assisted reproductive technologies such as IUI, IVF, and ICSI.^[5,21,25]^ However, these treatments still cannot meet the growing demand. The literature about exercise and male infertility increases in recent years, it is necessary to evaluate the impact of exercise on male infertility.

The ultimate treatment goal of male infertility is to address fertility problems and improve fertility. Therefore, this systematic review will use the 3 endpoints indicators of live-birth rate, pregnancy rate, adverse events (including miscarriage) to judge the therapeutic effect and safety of exercise on the reproductive outcomes of infertile male patients, regardless of whether the patients are combined with assisted reproductive treatment. And this research will only include male infertility populations. These above points are different from the previous evidence.^[14]^ Oligozoospermia, poor sperm morphology, and low sperm motility can lead to reduced fertilization rate.^[25]^ Increased sperm DNA fragmentation can cause spousal infertility, recurrent miscarriage, and affect the success rate of assisted reproductive technology.^[5]^ Therefore, we will also assess the effect of exercise on semen parameters including sperm concentration, sperm morphology, sperm forward motion, and sperm DNA fragmentation in infertile men.

In a word, this systematic review will provide evidence-based medical evidence whether exercise can improve the reproductive outcomes and reproductive capacity of male infertility, and provide guidance for clinicians’ decision making and patients’ lifestyle interventions. Moreover, further suggestions and ideas for further clinical studies will be made because we can understand the advantages and shortcomings of current clinical researches by this review.

This systematic review has the following limitations: First, due to the variety of sports, the search may not be comprehensive enough. Second, because of the unfamiliarity with other languages, the searched literature is limited to Chinese and English, which will cause certain bias. In addition, there may be a limited number and sample size of RCT for treating male infertility, the quality of evidence provided may not be high. Despite these limits, we still need to carry out this work to provide new suggestions for future researches on exercise treatment for male infertility.

### 3.1 Amendment

We changed the word “reproductive function” to “semen quality” in the title because the actual evaluation outcomes were “semen quality,” which was originally registered as “reproductive function.”

## Author contributions

**Conceptualization:** Xuhong Yan, Liang Dong, Degui Chang, Xujun Yu.

**Data curation:** Xuhong Yan, Liang Dong, Yinghong Liu.

**Formal analysis:** Xuhong Yan, Fang Yang, Kun Tan, Junjun Li.

Funding acquisition: Xujun Yu.

**Investigation:** Xuhong Yan, Liang Dong, Yinghong Liu, Fang Yang, Kun Tan, Junjun Li.

**Methodology:** Xuhong Yan, Liang Dong, Yinghong Liu.

**Project administration:** Xuhong Yan, Liang Dong, Degui Chang, Xujun Yu.

**Resources:** Degui Chang, Xujun Yu.

**Software:** Fang Yang, Kun Tan, Junjun Li.

**Supervision:** Xuhong Yan, Liang Dong, Yinghong Liu, Degui Chang, Xujun Yu.

**Validation:** Xuhong Yan, Fang Yang, Xujun Yu.

**Writing – original draft:** Xuhong Yan, Liang Dong, Yinghong Liu.

**Writing – review & editing:** Fang Yang, Kun Tan, Junjun Li, Degui Chang, Xujun Yu.
